# Supplementary material for: Physiological levels of 3-hydroxykynurenine alter mitochondrial function and morphology in neuronal cells
Source: bioRxiv. 2026 May 13:2026.05.13.724856. Preprint. [Version 1] doi: 10.64898/2026.05.13.724856 (PMC13192647; doi:10.64898/2026.05.13.724856)
Supplement: Supplement 1 [file media-1.pdf]

Supplementary data

Supplementary Figure 1: Characterisation of differentiated and undifferentiated SH-SY5Y cells. A) White light images of undifferentiated SH-SY5Y cells and cells differentiated for 11 days with 10 µM RA together with BDNF (12.5 ng/ml) for the final six days. Immunoblot analysis of (B) neuron specific enolase 2 and (C) MAP2 in undifferentiated and differentiated SH-SY5Y cells. (D) Representative images of undifferentiated SH-SY5Y cells following incubation with 3-HK (µM) for 24 hours. Mag = 20X. Scale bar = 100 µm.

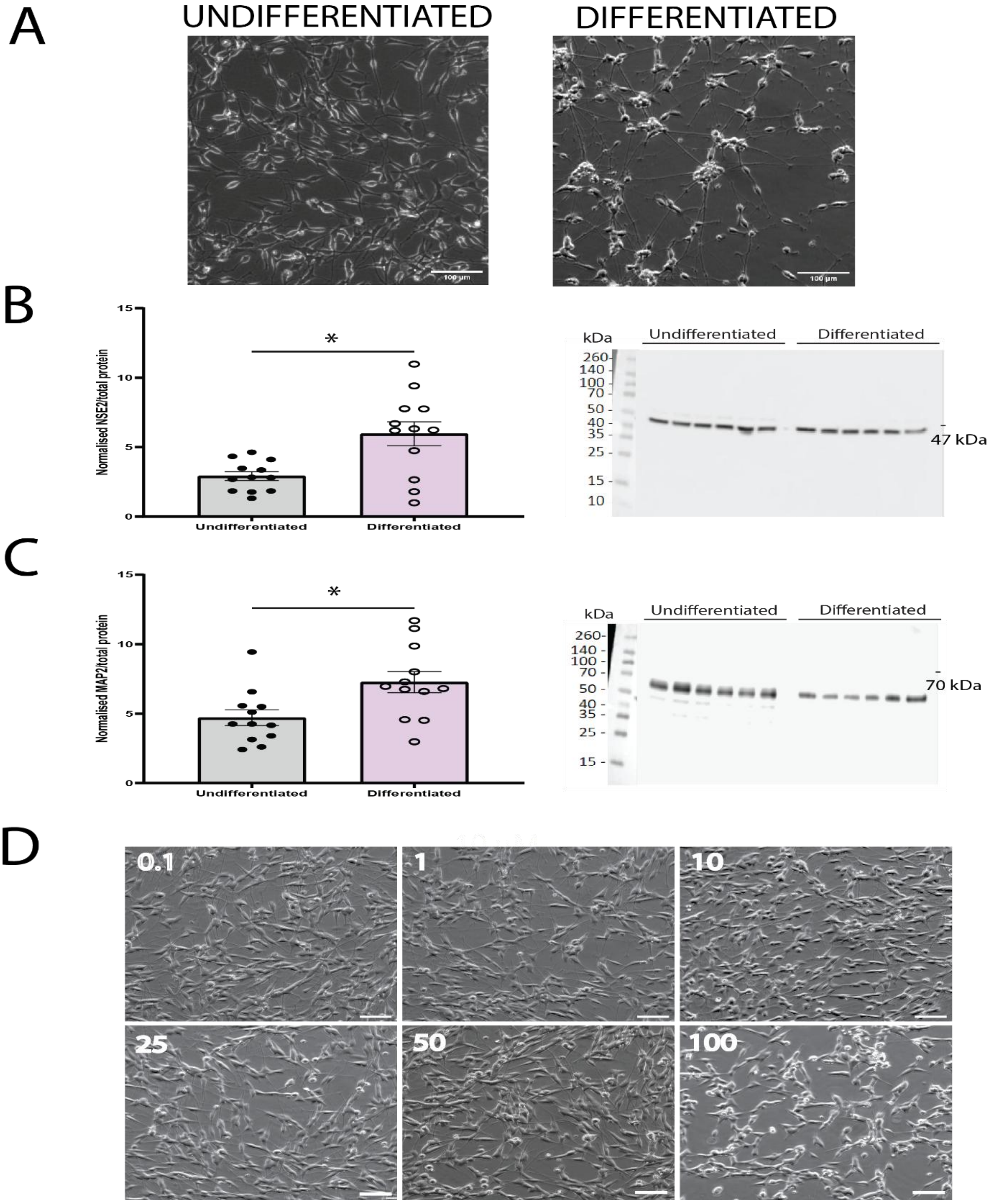

Supplementary Table 1: Kynurenine pathway and reference gene oligonucletides for qRT-PCR. Primers were purchased from Sigma-Aldrich. *3HAO = 3-hydroxyanthranilate oxidase. AADAT = Aminoadipate Aminotransferase. ACSMD = α-amino-β-carboxymuconate-ε-semialdehyde decarboxylase.* AFMID = arylformamidase. F = forward. IDO1 = *indoleamine 2,3-dioxygenase 1. IPO8 = importin 8. KMO = kynurenine 3-monooxygenase. CCBL1 = cysteine conjugate beta-lyase 1 (kynurenine aminotransferase 1). CCBL2 = cysteine conjugate beta-lyase 2 (kynurenine aminotransferase 3). KYNU = kynureninase. QPRT = Quinolinate phosphoribosyl transferase. qRT-PCR = quantitative reverse transcriptase polymerase chain reaction. R = reverse. Rplp0 = Ribosomal Protein Lateral Stalk Subunit P0. TDO2 = tryptophan 2,3-dioxygenase 2.*

Primer name	SEQUENCE (5'--> 3')	Description
hIDO1-F	GCCAGCTTCGAGAAAGAGTTG	Forward primer for hIDO1
hIDO1-R	ATCCCAGAACTAGACGTGCAA	Reverse primer for hIDO1
hTDO2-F	AAGGTTGTTTCTCGGATGCAC	Forward primer for hTDO2
hTDO2-R	TGTCATCGTCTCCAGAATGGAA	Reverse primer for hTDO2
hAFMID-F	TGGGTTTCCCAAGCAAGGTTC	Forward primer for hAFMID
hAFMID-R	TCTGCTCCCAGTCGGACAA	Reverse primer for hAFMID
hCCBL1-F	CAGACTTTGCCGTGGAAGCCTT	Forward primer for hCCBL1
hCCBL1-R	GCACATTCCTGAGCGGGTCTAT	Reverse primer for hCCBL1
hAADAT-F	TGTCACATCTGGCAGCCAACAAG	Forward primer for hAADAT
hAADAT-R	GAATAAGCAGGTTCATCTAGGAGG	Reverse primer for hAADAT
hCCBL2-F	GTAGTGCTCCACTTACACGAGG	Forward primer for hCCBL2
hCCBL2-R	GCTGCTTGAGTAACTGTCTCGAC	Reverse primer for hCCBL2
hKMO-F	GAATGCGGGCTTTGAAGAC	Forward primer for hKMO
hKMO-R	ACAGGAAGACACAAACTAAGGT	Reverse primer for hKMO
hKYNU-F	GTTGGCTTTGATCTAGCACATGC	Forward primer for hKYNU
hKYNU-R	TGAAGGCACCAGCAATTCCTCC	Reverse primer for hKYNU
h3-HAO-F	CCTGAGACAGAATGTGGACGTG	Forward primer for h3-HAO
h3-HAO-R	CTTGTGTTCGCTCCCAGGCATA	Reverse primer for h3-HAO
hQPRT-F	GTGAAGGATAACCATGTGGTGGC	Forward primer for hQPRT
hQPRT-R	CTGCTGCATTCCACTTCCACCT	Reverse primer for hQPRT
hACMSD-F	GGTGCGAGAGAATTGCTGG	Forward primer for hACMSD
hACMSD-R	TGCTGGCAAGGTCGTTGTTT	Reverse primer for hACMSD
hIPO8-F	AGGATCAGAGGACAGCACTGCA	Forward primer for hIPO8
hIPO8-R	AGGTGAAGCCTCCCTGTTGTTC	Reverse primer for hIPO8
hRPLP0-F	GCTGCTGCCCGTGCTGGTG	Forward primer for hRPLP0
hRPLP0-R	TGGTGCCCCTGGAGATTTTAGTGG	Reverse primer for hRPLP0

Supplementary Table 2: Kynurenine pathway enzymes constituently expressed in SH-SY5Y cells were detected using SYBR Green qRT-PCR. The assay was performed in triplicate. *NRT = reverse transcriptase null. AFMID = Arylformamidase. KYAT1 = Kynurenine aminotransferase 1. AADAT = Aminoadipate Aminotransferase. KYAT 3 = Kynurenine Aminotransferase 3. KMO = Kynurenine 3-Monooxygenase. IPO8 = importin 8. Rplp0 = Ribosomal Protein Lateral Stalk Subunit P0.*

**Gene**	**Cell Phenotype**	**Ct Value**	
		**Average**	**NRT**
**AFMID**	Non-differentiated	27.0	*Undetermined*
	Differentiated	24.5	*Undetermined*
**KYAT1**	Non-differentiated	27.5	*Undetermined*
	Differentiated	27.2	*Undetermined*
**AADAT**	Non-differentiated	26.1	*Undetermined*
	Differentiated	27.0	*Undetermined*
**KYAT3**	Non-differentiated	22.0	*Undetermined*
	Differentiated	24.3	*Undetermined*
**KMO**	Non-differentiated	34.2	35.9
	Differentiated	*Undetermined*	31.1
**IPO8**	Non-differentiated	27.0	*Undetermined*
	Differentiated	23.9	*Undetermined*
**RPLP0**	Non-differentiated	19.5	*Undetermined*
	Differentiated	21.7	*Undetermined*

Supplementary Figure 2: Treatment of SH-SY5Y cells with 10 µM 3-HK had no effect on mitochondrial respiration when measured as oxygen consumption rate (OCR: pmol/minute µg/mL of protein) using the Seahorse bioanalyzer XFp. (A) Representative graph of the Seahorse Bioenergetic Mito Stress test (Agilent Biosciences) in cells treated with 10 µM 3-HK and control cells. (B) Basal respiration, (C) ATP-linked respiration, (D) non-mitochondrial respiration and (E) coupling efficiency (%) (ATP-linked respiration rate/ basal respiration rate ×100) were quantified. Points represent the average value per replicate from 3 independent assays. Unpaired Student's t-test. Error bars indicate ± SEM.

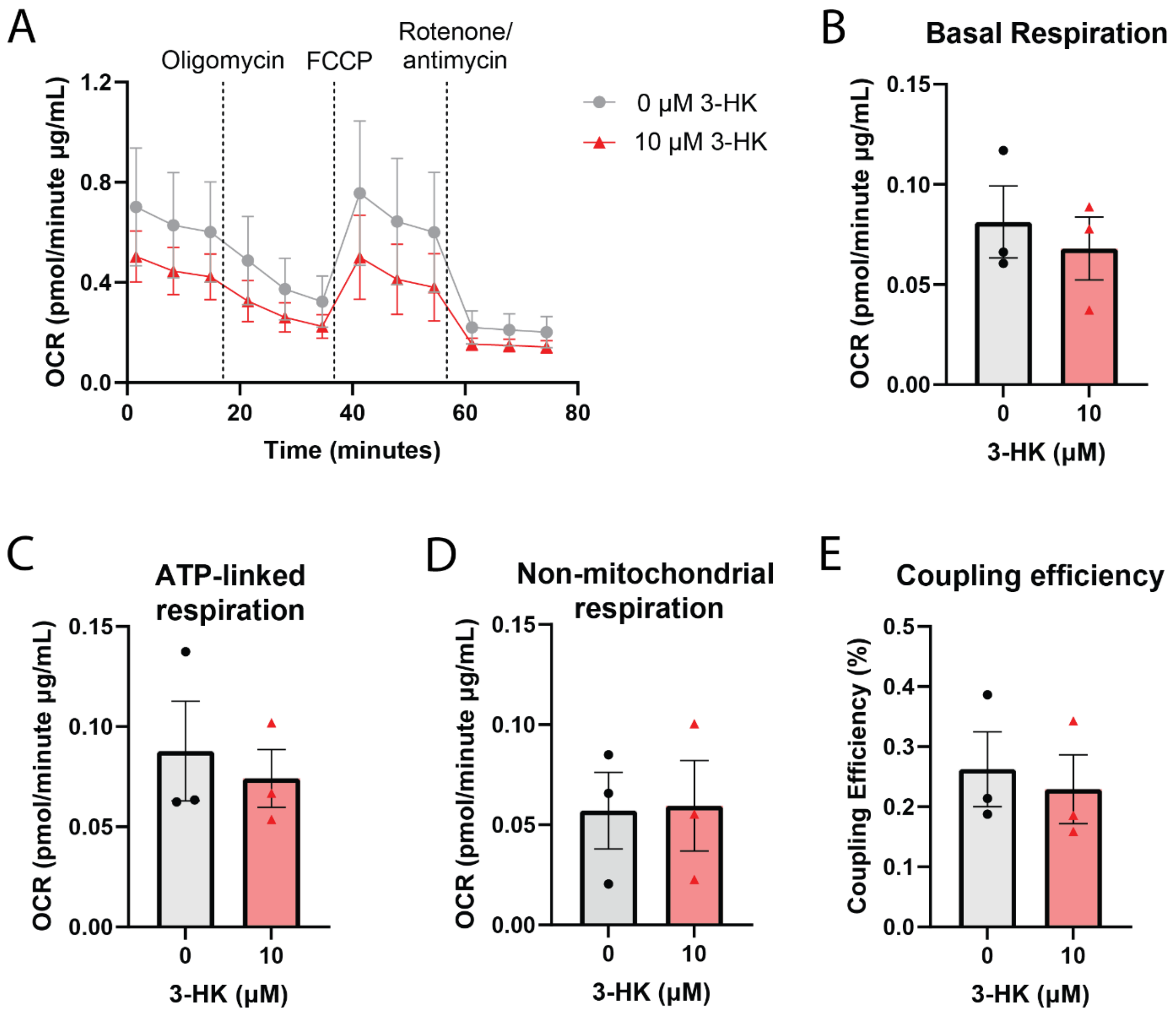

Supplementary Figure 3: The mitochondrial networks of SH-SY5Y cells were stained using Mitoview™ Green Dye and imaged by confocal microscopy for analysis following 24 hours exposure to 0, 0.1, 1 and 10 µM 3-HK. From maximum projection images: (A) The number of branches per mitochondria. (B) The average mitochondrial branch length (µm) per cell. Points represent a single cell and data is from 3 independent assays. Error bars indicate ± SEM.

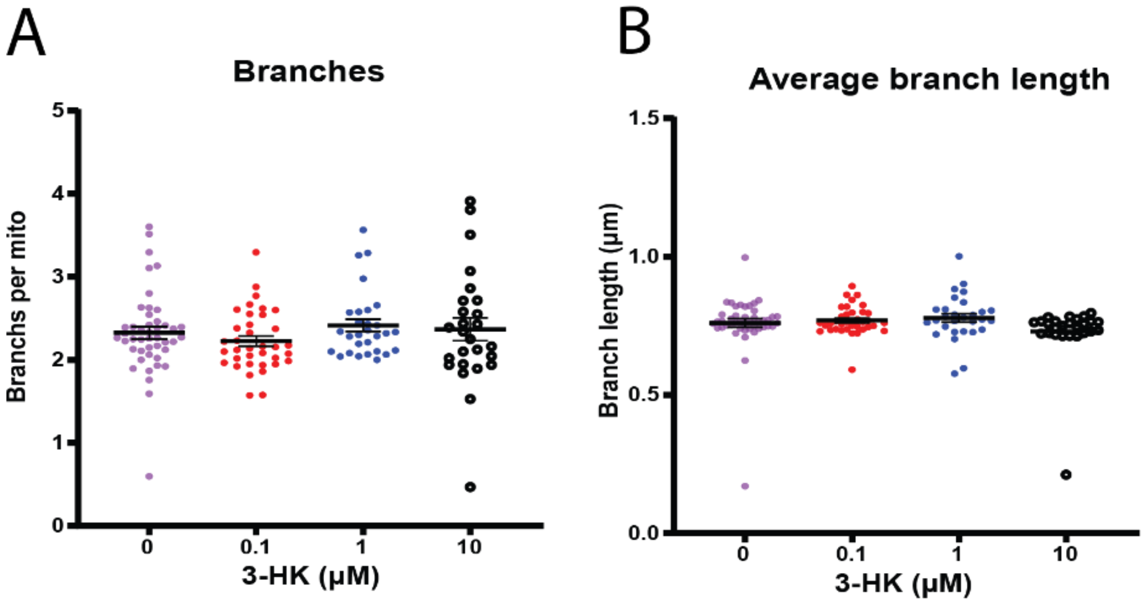

Supplementary Figure 4: F-actin staining of SH-SY5Y cells was unchanged following exogenous 3-HK treatment. Fluorescence intensity (AU) of Phalloidin-594 staining was measured via confocal microscopy to quantify F-actin. (A) No change in F-actin staining was observed at any tested concentration of 3-HK (µM) in comparison with the vehicle control. Points each represent a single cell and data is from 3 independent assays. One-way ANOVA. Error bars indicate ± SEM. (B) Representative images per condition. Magnification x100. Scale bar = 20 µm. *VEH = vehicle control.*

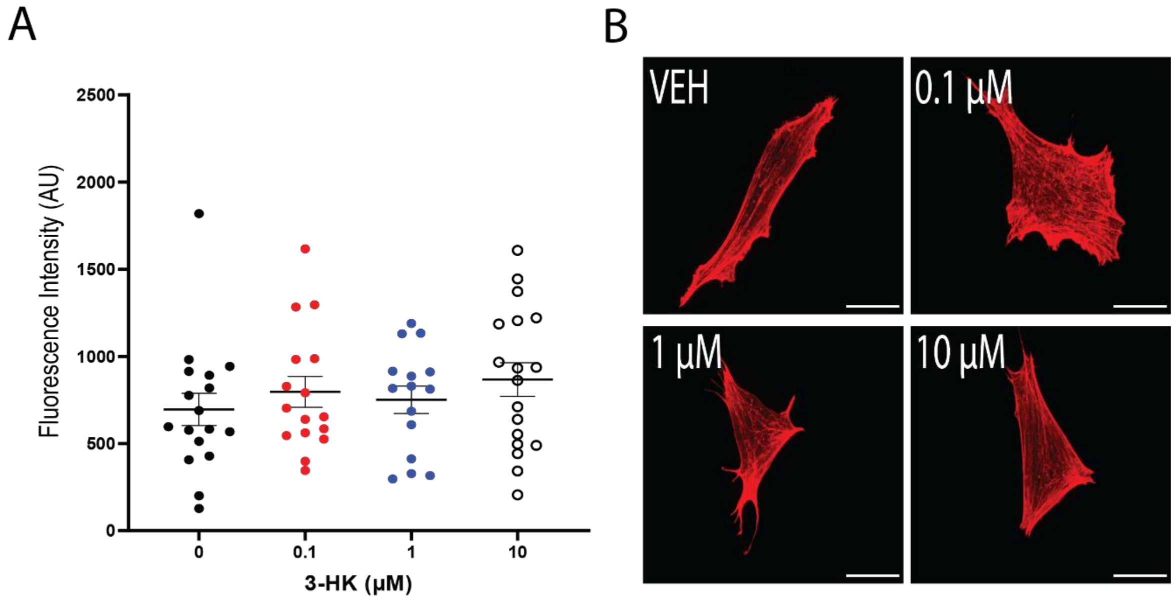

Supplementary Table 3: DESeq2 results table showing significantly up regulated differentially expressed transcripts (DETs) in each condition. Each transcript is presented by both its unique RefSeq transcript ID and the related common gene ID/symbol, where possible. Transcripts not mapped to a specific gene ID are shown as 'None'. The log2FoldChange (L2FC) and adjusted P-value (padj) are noted. Transcripts are ordered by descending log2FoldChange.

**UP REGULATED**			
**TRANSCRIPT ID**	**GENE SYMBOL**	**L2FC**	**PADJ**
**VEHICLE CONTROL VS 0.1 µM 3-HK**			
NM_001256270	KIF22	9.738339	0.001832
NM_024767	DLC1	7.997934	0.002549
NM_001278393	USP44	5.554421	2.28E-06
NM_001040110	NRF1	4.986878	0.005566
NM_001284338	NEDD4	4.256312	0.015145
NM_022963	FGFR4	3.956237	0.014643
NM_001204459	TNFRSF19	3.744702	0.030273
NM_001256163	BIRC2	3.358783	1.41E-06
NR_027489	MTHFSD	3.28713	0.006826
NM_001282585	MXRA8	2.672457	0.013677
NM_001166686	PFKM	2.629228	0.011187
NM_001161533	None	2.441415	0.00011
NM_001163436	TBCK	2.352795	0.003614
NR_105052	PSD2-AS1	2.038559	0.035616
NM_015493	KANK2	1.992757	0.002107
NM_001191324	RNF138	1.859071	0.042656
NM_001254749	RGS5	1.691107	2.45E-05
NM_001009812	LBX2	1.664739	0.038129
NM_001256879	POLD2	1.593209	0.03099
NM_032853	PWWP3A	1.513908	1.71E-07
NM_001042595	TMEM91	1.429053	0.018702
NM_134426	SLC26A6	1.368685	0.036484
NM_001097594	XAGE1A	1.329517	0.015363
NR_027167	SNHG29	1.289702	0.004533
NM_001040454	SLC26A6	1.244248	0.006826
NM_004772	NREP	1.201608	8.92E-06
NM_001286672	AASDH	1.110469	0.002512
NR_130944	WDR73	1.081557	0.02321
NM_001080427	None	1.008411	0.007821
NM_003250	THRA	0.930419	0.007246
NR_027504	MST1P2	0.814836	0.009405
NM_006531	IFT88	0.810251	0.002496
NR_002190	SUMO1P3	0.798795	0.017907
NR_024166	ZNF205-AS1	0.792977	0.04904
NR_104307	ULK3	0.773158	0.02042
NM_002982	CCL2	0.686678	0.020731
NM_001098577	RPL31	0.653554	0.008703
NR_038879	PAXBP1-AS1	0.632831	0.013722
NM_003260	TLE2	0.572646	0.004957
**VEHICLE CONTROL VS 1 µM 3-HK**			
NM_033668	ITGB1	6.920805	0.000833
NM_001284274	HECTD2	5.748252	0.019864
NM_001164824	SMIM12	5.538854	0.001602
NM_000704	ATP4A	5.465517	0.002114
NR_040712	None	5.280932	0.015731

NR_024125	ATP1A1-AS1	4.796088	0.028071
NM_001261438	ETV4	4.623017	0.004446
NM_001297778	NMNAT1	4.473952	0.000337
NM_001282660	MRTFA	4.391425	4.61E-16
NM_001303108	PGAP4	4.125634	0.01968
NM_001287044	VEGFA	3.995145	0.021994
NM_001079877	RASA4	3.901976	4.36E-05
NM_174963	ST3GAL3	3.764657	0.000381
NM_001166686	PFKM	3.011991	0.01281
NR_002715	RN7SL1	2.979518	0.003567
NM_001282568	ZCCHC17	2.751469	0.013743
NM_001242701	CAMTA1	2.695539	0.005622
NM_001009812	LBX2	2.181117	0.011696
NR_027260	RN7SL2	2.174626	0.039876
NM_001454	FOXJ1	2.026516	0.004243
NM_174971	ST3GAL3	2.011957	0.030466
NM_022805	SNRPN	1.592899	0.029847
NM_199191	BABAM2	1.440381	0.001472
NM_003099	SNX1	1.325315	0.021607
NM_001291018	PDE6D	1.308704	0.043573
NR_029390	None	1.248058	0.016125
NR_110373	LINC01159	1.167409	0.02754
NM_001012973	PLAC9	0.895829	0.045922
NM_003260	TLE2	0.730584	0.002063
NR_036487	FGF14-AS2	0.563224	0.015484
**VEHICLE CONTROL VS 10 µM 3-HK**			
NM_001102664	EPN2	9.92656	0.001089
NM_015350	LRRC8B	5.90399	0.00944
NM_017787	WBP1L	5.698395	0.036647
NM_001282886	EIF4E3	5.574405	0.000974
NM_001278393	USP44	5.528973	3.01E-06
NM_001199014	STPG1	4.892734	0.000981
NR_003286_2	None	4.779938	0.001333
NM_001100603	KDELR2	4.512632	0.038057
NM_001271960	SLC39A1	4.460067	0.001714
NM_001290307	CTNNA1	4.046996	0.037501
NM_001174097	LDHB	3.696275	0.031055
NM_001256335	PTGES2	3.595795	2.73E-05
NM_015520	MAGI1	3.269136	0.01912
NM_001257361	AMPD2	3.112858	0.003104
NM_001282568	ZCCHC17	2.807842	0.002353
NM_001286109	CLN3	2.4998	0.016657
NM_001307951	LPXN	2.33095	0.046679
NM_032853	PWWP3A	2.274606	7.40E-16
NM_001009812	LBX2	2.245881	0.002176
NM_003169	SUPT5H	2.076136	0.003114
NM_006317	BASP1	1.858747	0.000635
NM_001080427	None	1.81309	4.12E-08
NM_001243204	ECSIT	1.715635	0.040649
NM_001161533	None	1.650452	0.023048
NR_046096	CPEB1-AS1	1.62884	0.017078
NR_110542	PIK3IP1-DT	1.598313	0.007181
NM_172095	CATSPER2	1.40655	1.58E-05
NM_001243738	RGL2	1.353189	0.004706

NM_001282583	MXRA8	1.296908	0.013626
NR_003574	ABCA17P	1.251936	0.007086
NM_020877	DNAH2	1.217342	0.00842
NM_001282497	ZNF343	1.097727	0.0313
NR_110220	LINC01237	0.979032	0.048142
NM_022468	MMP25	0.883683	0.037778
NM_014741	ATG13	0.761813	0.04611
NM_015833	ADARB1	0.753944	0.022722
NM_001300927	PGPEP1	0.62556	0.048542
NM_001282494_1	None	0.59094	0.003898
**0.1 μM 3-HK VS 1 μM 3-HK**			
NM_001277335	RASA4B	14.66796	8.81E-12
NM_052985	IFT122	9.89781	3.38E-05
NM_001204887	RAB43	9.408757	0.001838
NM_001303108	PGAP4	7.189597	2.52E-05
NM_001284274	HECTD2	6.6406	0.00777
NM_033668	ITGB1	6.479809	0.001687
NM_001164824	SMIM12	6.022465	0.000528
NR_040712	CHRAC1	5.12465	0.01854
NM_001297778	NMNAT1	4.494715	0.0003
NM_001282660	MRTFA	4.387944	4.92E-16
NM_001206929	AGER	4.188927	0.03536
NM_001261438	ETV4	3.718227	0.02305
NM_174963	ST3GAL3	3.652346	0.000546
NM_001278205	None	3.589867	0.024454
NM_201595	GTF2A1	3.574857	0.025728
NM_001142310	TMEM169	3.153783	0.029249
NM_001170580	HHAT	3.07133	0.027967
NM_174971	ST3GAL3	2.963488	0.00093
NM_001173482	CRBN	2.942856	0.03165
NR_001445	RN7SK	2.82016	0.030828
NM_001273	CHD4	2.785769	5.42E-08
NR_002715	RN7SL1	2.591334	0.010741
NR_002569	SCARNA9	2.471803	0.003653
NM_001013406	KRIT1	2.369748	0.014035
NM_020734	RIMKLB	2.26333	0.02322
NM_001105540	DGKZ	1.9011	0.000196
NM_003455	ZNF202	1.884836	1.44E-05
NM_001813	CENPE	1.801537	0.033902
NM_001205179	ALKBH2	1.781041	0.014898
NM_001168222	TBC1D17	1.705659	0.009661
NM_001282652	STIP1	1.645486	0.006908
NM_001278189	TPM3	1.588036	7.01E-07
NM_052870	SNX18	1.575437	0.00898
NM_001454	FOXJ1	1.525945	0.029821
NM_003099	SNX1	1.410926	0.012915
NR_033489	ABHD16A	1.202027	0.026276
NR_051960	FALEC	1.090007	0.039852
NM_001286582	PHRF1	1.079649	0.047857
NM_001024809	RARA	1.019037	0.010329
NM_001267783	AMBRA1	0.909821	0.001532
NR_073599	None	0.893083	0.005928
NM_181727	SPATA12	0.840312	0.038043
NM_012237	SIRT2	0.712758	0.00898

NM_024033	CYREN	0.682922	0.023726
**0.1 µM 3-HK VS 10 µM 3-HK**			
NM_001277335	RASA4B	14.6841	3.75E-15
NM_017787	WBP1L	11.43479	0.000126
NM_001098493	ZNF419	10.10302	2.22E-07
NM_001098496	ZNF419	9.618508	1.68E-06
NR_103455	ZSCAN16-AS1	8.910362	3.00E-07
NM_001277947	ZNF83	7.941939	0.00268
NM_178173	IHO1	6.298739	0.01756
NR_003286_2	None	5.049326	0.000478
NM_001172225	ZNF540	4.869123	0.018021
NM_001303108	PGAP4	4.846815	0.000821
NM_001278926	PPWD1	4.553677	0.033
NM_202758	ENOSF1	4.5324	0.016696
NM_001193369	DIDO1	3.920519	0.0001
NR_040053	RNF41	3.759373	0.000511
NM_001197079	IFRD1	3.694166	0.02182
NM_001282719	LDAH	3.619675	0.004744
NM_001267818	OSTC	3.607314	0.000688
NR_033836	PIGA	3.602479	0.008018
NM_001278205	None	3.219562	0.018096
NM_001173482	CRBN	3.20516	0.004686
NR_037649	CCNT2	3.181526	0.007023
NM_015520	MAGI1	3.131458	0.02244
NM_001145548	ZDHHC7	3.089417	0.020209
NM_001271960	SLC39A1	2.986281	0.044194
NM_153047	FYN	2.802812	0.010196
NM_181527	NAA20	2.557755	0.01316
NM_001813	CENPE	2.493094	0.000205
NM_001145287	OPRM1	2.456373	0.007175
NM_001273	CHD4	2.413364	5.37E-09
NM_001256335	PTGES2	2.105594	0.025784
NM_001160390	TRPT1	1.996691	0.006643
NM_001205179	ALKBH2	1.98907	0.000992
NM_001105540	DGKZ	1.9581	1.38E-06
NM_006317	BASP1	1.848278	0.000588
NM_001278189	TPM3	1.656047	8.01E-11
NM_003169	SUPT5H	1.63318	0.026997
NM_001243738	RGL2	1.539372	0.000694
NR_034121	CKMT2-AS1	1.403649	0.042249
NM_001145011	C16orf96	1.17382	0.044602
NM_001204368	MGST2	1.167481	0.038044
NR_120577	ESAM-AS1	1.091333	0.000932
NR_110220	LINC01237	1.012859	0.034283
NM_001282723	LDAH	1.003243	0.017433
NR_026974	ZNF252P-AS1	0.95234	0.030937
NM_199334	THRA	0.946991	0.001362
NM_002285	AFF3	0.892093	0.030675
NM_198395	G3BP1	0.843925	0.007297
NM_001285450	PDXDC1	0.813798	0.030937
NM_001301072	MAP3K4	0.802075	0.038854
NM_012237	SIRT2	0.783508	0.000416
NM_002941	ROBO1	0.730228	0.000716
NM_001204747	RFC1	0.721332	0.014222

NR_002473	None	0.678943	0.023574
NR_040013	LOC644554	0.610662	0.013787
**1 μM 3-HK VS 10 μM 3-HK**			
NM_001135651	EIF2AK2	14.31435	9.61E-11
NM_024772	ZMYM1	13.10537	1.32E-14
NM_001194955	MATR3	11.39406	0.000107
NM_001199014	STPG1	10.5672	2.06E-05
NR_040762	ZIC4	9.613276	2.58E-07
NM_001310339	MGME1	9.535294	5.87E-07
NR_033836	PIGA	9.16116	8.27E-05
NM_194454	KRIT1	8.439637	1.55E-14
NM_006661	PDE10A	8.185792	3.19E-07
NM_001098496	ZNF419	8.142011	0.000895
NM_001172225	ZNF540	7.799394	0.006933
NM_005520	HNRNPH1	6.839359	7.02E-07
NM_001288632	FAM222B	6.833451	0.00123
NM_014432	IL20RA	6.562071	6.23E-08
NM_024515	WDR25	5.88177	4.41E-05
NM_175735	LYG2	5.632041	0.002641
NR_110156	LINC01798	5.495469	0.005721
NR_003286_2	None	5.481979	0.001656
NM_148955	SNX1	5.393912	5.02E-08
NM_001098493	ZNF419	5.33323	0.004464
NM_001206799	PKM	5.117624	2.00E-05
NM_001164747	RASSF8	4.999121	0.00071
NM_001204171	MDM4	4.682813	1.54E-10
NM_017996	DET1	4.24968	6.56E-07
NM_005734	HIPK3	4.191239	2.71E-05
NM_181482	LDLRAD4	4.081661	0.000714
NR_040585	STAG3L4	4.048867	0.001441
NM_001256335	PTGES2	3.694882	0.000656
NM_004361	CDH7	3.626062	0.000432
NM_001286837	RNASEH1	3.02481	0.000825
NM_001278174	ZNF33A	2.955388	0.020557
NM_001290259	PHF3	2.903828	0.003864
NM_001178011	CDC45	2.865359	0.002805
NM_001085377	MCC	2.778641	0.023358
NM_001142327	DMTF1	2.778022	0.002109
NM_001005741	GBA	2.593427	1.82E-05
NR_125792	LINC01291	2.500448	0.038646
NM_001114617	MGAT1	2.391266	5.30E-06
NM_001243374	CLCN3	2.365504	0.009235
NM_203401	STMN1	2.270622	0.028262
NM_197966	BID	2.227611	2.80E-05
NM_001130849	CAB39	2.211567	0.042984
NM_001134774	KLC2	2.198945	0.001911
NM_001286589	AIG1	2.178339	0.007888
NM_024731	KLHL36	2.048586	0.003977
NR_024514	ADAMTS13	2.00693	0.049828
NM_001170780	PABIR3	1.936016	0.03442
NM_006317	BASP1	1.857437	0.005074
NM_032853	PWWP3A	1.787955	5.87E-07
NM_203505	G3BP2	1.775836	0.005852
NM_000093	COL5A1	1.739793	4.77E-05

NM_022372	MLST8	1.685967	0.011742
NM_001301819	ZNF202	1.676746	6.60E-06
NM_003671	CDC14B	1.674478	0.008302
NM_003169	SUPT5H	1.670962	0.037517
NM_012472	DNAAF11	1.598646	0.000522
NM_001014	RPS10	1.570797	0.008306
NM_001455	FOXO3	1.511315	0.025897
NM_031501	PCDHA5	1.316989	0.012225
NM_001199746	HOXD8	1.284557	0.008117
NM_000333	ATXN7	1.253063	0.042479
NM_138559	BCL11A	1.219127	0.02651
NR_125729	LINC00680	1.196582	0.014243
NR_110099	SNHG21	1.190132	0.010027
NM_024867	SPEF2	1.179244	0.009566
NM_022107	GPSM3	1.178587	0.002272
NR_073395	FAM90A25P	1.172752	0.014941
NM_018850	ABCB4	1.162026	0.002582
NM_001076785	SLC7A6	1.120457	0.021053
NM_024877	CCNP	1.109266	0.047639
NM_001080427	None	1.094523	0.008484
NM_180699	SNRNP35	1.081142	0.021874
NM_001285450	PDXDC1	1.070917	0.01132
NM_001267574	EIF3C	1.048598	0.005966
NM_021038	MBNL1	1.035235	0.032834
NR_122109	CYP51A1-AS1	1.034508	0.030416
NM_001127361	RNF19B	1.009333	0.041696
NM_001172677	ZNF607	0.990643	0.007678
NM_001199355	RPL17-C18orf32	0.979912	0.008693
NM_005888	SLC25A3	0.969299	0.013333
NM_001278217	CDK5RAP3	0.94933	0.04678
NM_022757	CCDC14	0.923131	0.043744
NM_020831	MRTFA	0.90457	1.40E-07
NR_040013	LOC644554	0.871154	0.003091
NM_007222	ZHX1	0.819201	0.009146
NM_001668	ARNT	0.739339	0.036353
NM_153255	MCM9	0.685808	0.042258
NM_030674	SLC38A1	0.669886	0.020317
NM_032926	TCEAL3	0.666639	0.003534
NM_014884	SUGP2	0.654387	0.005024

Supplementary Table 4: DESeq2 results table showing significantly down regulated differentially expressed transcripts (DETs) in each condition. Each transcript is presented by both its unique RefSeq transcript ID and the related common gene ID/symbol, where possible. Transcripts not mapped to a specific gene ID are shown as 'None'. The log2FoldChange (L2FC) and adjusted P-value (padj) are noted. Transcripts are ordered by descending log2FoldChange.

**DOWN REGULATED**			
**TRANSCRIPT ID**	**GENE SYMBOL**	**L2FC**	**PADJ**
**VEHICLE CONTROL VS 0.1 µM 3-HK**			
NM_134263	SLC26A6	-0.56924	0.007302
NM_001278463	DNM1L	-0.66023	0.035616
NM_002693	POLG	-0.68101	0.044335
NR_073599	None	-0.71123	0.013492
NM_022911	SLC26A6	-0.74807	0.000371
NM_001204747	RFC1	-0.76589	0.008655
NM_012237	SIRT2	-0.8089	0.000242
NM_199334	THRA	-0.8894	0.003425
NM_001278189	TPM3	-0.88945	0.001279
NM_001273	CHD4	-1.80568	2.19E-05
NR_110927	SVIL-AS1	-2.15756	0.004688
NM_001813	CENPE	-2.41438	0.000368
NM_001100913	PACS2	-2.43777	0.02505
**VEHICLE CONTROL VS 1 µM 3-HK**			
NM_001136472	LITAF	-0.55277	0.008945
NM_001024924	EXOC1	-0.57278	0.032852
NM_001098616	C1orf43	-0.58819	0.029885
NM_001015048	BAG5	-0.59052	0.048427
NM_144669	GLT1D1	-0.63635	0.004494
NM_012161	FBXL5	-0.65816	0.049676
NM_031450	C11orf68	-0.74016	0.004113
NM_001164380	STAU2	-0.74575	0.018124
NM_001305155	PPP2R3C	-0.78013	0.044034
NM_001191005	SRSF10	-0.82913	0.024847
NM_017958	PLEKHB2	-0.85581	0.026471
NM_001668	ARNT	-0.87793	0.011717
NM_020831	MRTFA	-0.92071	6.00E-08
NM_013235	DROSHA	-0.93972	0.037395
NM_006444	SMC2	-0.99149	0.022238
NM_001172677	ZNF607	-1.00123	0.006754
NR_024111	SBDSP1	-1.00543	0.005025
NM_001076785	SLC7A6	-1.07628	0.02513
NM_005097	LGI1	-1.13666	0.004251
NM_180699	SNRNP35	-1.22635	0.008974
NM_001310332	RNF31	-1.28485	7.23E-05
NM_001199355	RPL17-C18orf32	-1.30288	0.000391
NM_001301819	ZNF202	-1.38181	0.000243
NM_000093	COL5A1	-1.643	0.000137
NM_024772	ZMYM1	-13.5351	1.63E-14
**VEHICLE CONTROL VS 10 µM 3-HK**			
NR_024111	SBDSP1	-0.87275	0.0046
NR_045116	None	-0.88108	0.00944
NM_001310332	RNF31	-1.25675	8.98E-07
NR_024247	PWWP3A	-1.35417	1.29E-08
NM_031449	ZMIZ2	-1.5674	0.007655

NR_125341	DDX46	-2.34708	0.000236
NM_001206956	CNTN4	-2.58141	2.74E-06
NM_001255986	COLEC11	-2.72058	0.012338
**0.1 µM 3-HK VS 1 µM 3-HK**			
NM_001258217	MIS12	-0.55743	0.030625
NM_032926	TCEAL3	-0.56567	0.011511
NM_031450	C11orf68	-0.71664	0.00512
NM_145276	ZNF563	-0.77244	0.006557
NM_020831	MRTFA	-0.83415	1.16E-06
NM_001244813	FOXP1	-0.83693	0.032528
NM_001267574	EIF3C	-0.91665	0.014686
NM_001199267	DGKZ	-0.94507	0.030189
NR_110801	LOC100507002	-0.95006	0.02239
NM_032853	PWWP3A	-0.95889	0.00884
NM_004772	NREP	-0.96608	0.004529
NM_001164840	LYRM4	-0.98332	0.007484
NM_001145468	SPECC1L	-1.00764	0.04471
NM_001310332	RNF31	-1.00779	0.002071
NM_022107	GPSM3	-1.04824	0.006132
NM_001128226	DIS3	-1.06	0.021007
NM_001301856	ELOVL5	-1.06393	0.034458
NM_001271951	TPGS2	-1.13824	0.002283
NM_000093	COL5A1	-1.1624	0.006849
NM_001172677	ZNF607	-1.1928	0.001049
NM_001297553	CHD4	-1.20487	0.007721
NM_003205	TCF12	-1.38335	0.015603
NM_003646	DGKZ	-1.47033	0.017771
NM_001199746	HOXD8	-1.48397	0.001904
NM_001287603	ZBTB17	-1.49822	0.034979
NM_012472	DNAAF11	-1.57875	0.000538
NM_001254749	RGS5	-1.59929	0.001229
NM_001199355	RPL17-C18orf32	-1.6012	9.26E-06
NM_001301819	ZNF202	-1.63701	1.02E-05
NM_001014	RPS10	-1.73195	0.003079
NM_197966	BID	-1.95284	0.000233
NM_001278174	ZNF33A	-3.00301	0.017528
NM_024772	ZMYM1	-13.5906	7.50E-15
**0.1 µM 3-HK VS 10 µM 3-HK**			
NM_024307	GDPD3	-0.56107	0.002675
NR_002450	SNORD68	-0.60962	0.037857
NR_046325	PCAT6	-0.60994	0.016142
NR_024247	PWWP3A	-0.67704	0.01256
NM_001105659	LRRIQ3	-0.7142	0.016433
NM_001003788	STRADA	-0.79853	0.041356
NM_003250	THRA	-0.83323	0.018595
NR_038378	LOC441242	-0.92044	0.006438
NM_003432	ZNF131	-0.92561	0.02602
NM_002982	CCL2	-0.94799	0.000416
NM_004772	NREP	-0.95447	0.000619
NM_001310332	RNF31	-0.9925	0.000154
NM_015493	KANK2	-1.73523	0.008761
NM_013274	POLL	-1.74004	0.019582
NR_130727	LOC401357	-1.80657	0.00055
NM_003646	DGKZ	-1.85346	0.000286

NM_001254749	RGS5	-1.88052	1.91E-06
NM_001256163	BIRC2	-2.19009	0.002988
**1 µM 3-HK VS 10 µM 3-HK**			
NM_003099	SNX1	-1.27184	0.026831
NR_024247	PWWP3A	-1.36091	6.60E-06
NR_130727	LOC401357	-1.54988	0.012869
NM_001079877	RASA4	-2.67497	0.006277
NM_001242701	CAMTA1	-2.83485	0.003476
NM_174971	ST3GAL3	-3.21388	0.000369
NR_024176	MKNK1	-3.45816	0.006483
NM_001297778	NMNAT1	-3.81124	0.002374
NM_001282660	MRTFA	-3.89904	6.22E-13
NM_001164824	SMIM12	-5.47745	0.00168

Supplementary Figure 5: Significant differentially expressed transcripts (DETs) per condition presented as Venn diagrams to highlight the number of overlapping DETs between experimental conditions. Data does not discriminate between up regulated or down regulated DETs. (A) Overlapping DETs for 0.1, 1 and 10 µM 3-HK vs vehicle control (0.02% DMSO). (B) Overlapping DETs for 0.1, 1 and 10 µM 3-HK compared to each other.

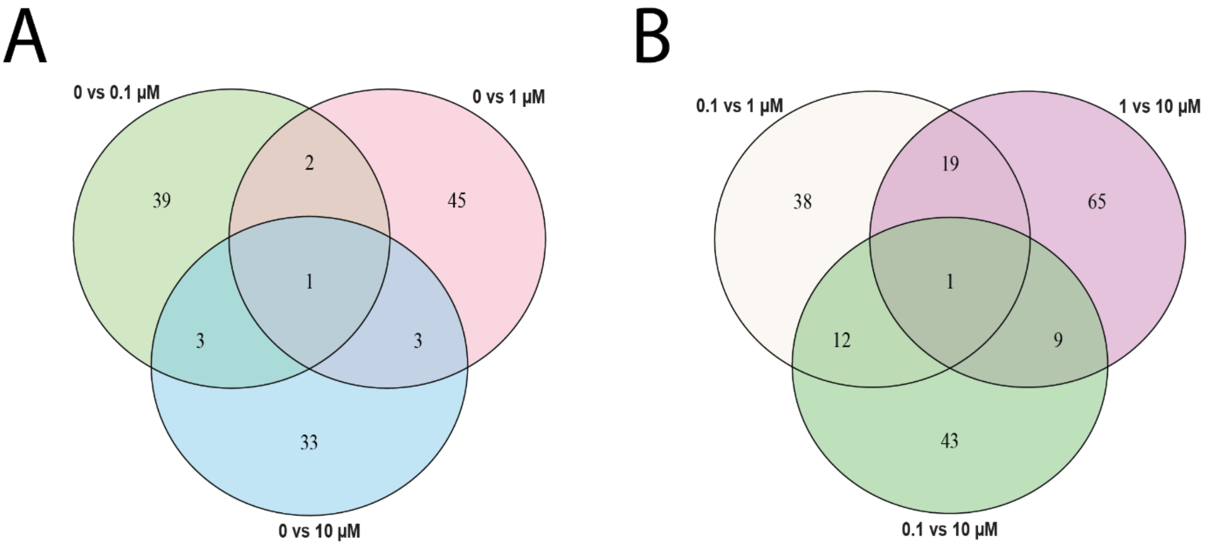

Supplementary Table 5: Table presenting the transcript and related gene names of significantly differentially expressed transcripts (DETs) which are up/down regulated in more than one experimental condition. Data does not discriminate between up regulated or down regulated DETs.

TRANSCRIPT ID	GENE SYMBOL
**VEHICLE CONTROL vs 0.1 µM 3-HK VS VEHICLE CONTROL vs 1 µM 3-HK**	
NM_001166686	PFKM
NM_003260	TLE2
**VEHICLE CONTROL vs 0.1 µM 3-HK VS VEHICLE CONTROL vs 10 µM 3-HK**	
NM_001282585	MXRA8
NM_032853	PWWP3A
NM_001278393	USP44
**VEHICLE CONTROL vs 1 µM 3-HK VS VEHICLE CONTROL vs 10 µM 3-HK**	
NM_001310332	RNF31
NR_024111	SBDSP1
NM_001282568	ZCCHC17
**VEHICLE CONTROL vs 0.1 µM 3-HK VS VEHICLE CONTROL vs 1 µM 3-HK VS VEHICLE CONTROL vs 10 µM 3-HK**	
NM_001009812	LBX2
**0.1 µM 3-HK vs 1 µM 3-HK VS 0.1 µM 3-HK vs 10 µM 3-HK**	
NM_001205179	ALKBH2
NM_001813	CENPE
NM_001273 & NM_001297553	CHD4
NM_001173482	CRBN
NM_001105540 & NM_003646 & NM_001199267	DGKZ
NM_004772	NREP
NM_001303108	PGAP4
NM_001277335	RASA4B
NM_001254749	RGS5
NM_001310332	RNF31
NM_012237	SIRT2
NM_001278189	TPM3
**0.1 µM 3-HK vs 1 µM 3-HK VS 1 µM 3-HK vs 10 µM 3-HK**	
NM_197966	BID
NM_000093	COL5A1
NM_012472	DNAAF11
NM_001267574	EIF3C
NM_022107	GPSM3
NM_001199746	HOXD8
NM_001013406 & NM_194454	KRIT1
NM_001282660 & NM_020831	MRTFA
NM_001297778	NMNAT1
NM_001199355	RPL17-C18orf32
NM_001014	RPS10
NM_001164824	SMIM12
NM_148955 & NM_003099	SNX1
NM_174971 & NM_174963	ST3GAL3
NM_032926	TCEAL3
NM_024772	ZMYM1
NM_003455 & NM_001301819	ZNF202
NM_001278174	ZNF33A
NM_001172677	ZNF607
**0.1 µM 3-HK vs 10 µM 3-HK VS 1 µM 3-HK vs 10 µM 3-HK**	
NM_006317	BASP1

NR_130727	LOC401357
NR_040013	LOC644554
NM_001285450	PDXDC1
NR_033836	PIGA
NM_001256335	PTGES2
NM_003169	SUPT5H
NM_001098493 & NM_001098496	ZNF419
NM_001172225	ZNF540
**0.1 µM 3-HK vs 1 µM 3-HK VS 0.1 µM 3-HK vs 10 µM 3-HK VS 1 µM 3-HK vs 10 µM 3-HK**	
NM_032853 & NR_024247	PWWP3
